# The Need for a Unified Protocol for Termination of Amblyopia Treatment

**DOI:** 10.22599/bioj.109

**Published:** 2018-04-24

**Authors:** Mahmoud M. Nassar, Fiona Campbell Mitchell

**Affiliations:** 1 Ophthalmology Minia University Hospital, EG; 2 NHS Dumfries and Galloway, GB

**Keywords:** amblyopia, amblyopia recurrence, amblyopia treatment, visual regression

## Abstract

**Introduction:**

Many authors have investigated the methodology and outcomes of amblyopia treatment. However, the evidence on termination of treatment, specifically referring to the stability of visual outcome and timing of reviewing patients after treatment ends, has received less interest with no agreement on risk factors of visual regression.

**Purpose:**

To study the final part of amblyopia service with particular emphasis to stability of visual outcome; efficacy and timing of follow up after treatment ends.

**Methods:**

A retrospective review of patients discharged following treatment for strabismic and anisometropic amblyopia. Exclusion criteria were ocular pathology, poor attendance or poor compliance. Collected data included: age at the start of treatment, type and duration of treatment, number of visits, visual acuity (at start and end). Additionally, we analysed the duration and number of visits after stopping treatment, final visual acuity at discharge and duration of treatment with minimal change in vision.

**Results:**

Thirty-nine patients were identified with a mean age 4.2 ± 1.7SD years. Patients had an average of 116.6 ± 13.9SD visits over 30.5 ± 21.5SD months. Of these, 71.8% had occlusion only and 28.2% in combination with atropine. All but three patients had improved vision, with mean letters gained 13.2 ± 8.7SD LogMAR. The improvement in vision was statistically significant p < 0.005 and patients were further followed without treatment for 10 ± 13.5SD months over 5.2 ± 6.6SD visits. The mean final vision at discharge was not significantly different from vision at termination (p = 0.68) and we found no significant recurrence.

**Conclusion:**

Amblyopia treatment is effective; however, there is inconsistency in many aspects of ending treatment. Further improvement is needed to standardise care from termination of treatment up to discharge from service.

## Introduction

Many authors have intensely investigated the management and outcomes of amblyopia treatment. Despite the fact that recurrence of amblyopia following cessation of treatment is expected there is no agreement on the exact incidence or the risk factors associated with visual regression (Tacagni et al. 2007; Gunton 2013; Nilsson, Baumann and Sjöstrand 2007).

Current practice consists of an evidence-based approach to diagnose and treat amblyopia; however, the protocols for the cessation of treatment and duration of post-treatment follow-up remain arbitrary (Tacagni et al. 2007). Previous studies have suggested that maximal visual acuity is reached by 15 weeks of treatment and visual regression can occur in up to 67% following cessation of treatment (Gunton 2013), and usually within six months post-treatment (Nilsson, Baumann and Sjöstrand 2007). The published risk factors include the presence of microtropia, age at start of treatment, abrupt cessation of treatment, the amount of improvement in visual acuity, age at termination of amblyopia treatment and the final visual acuity (Tacagni et al. 2007; Gunton 2013; Nilsson, Baumann and Sjöstrand 2007; Saxena et al. 2013; Walsh et al. 2009; Holmes et al. 2007; Bhola et al. 2006; Pediatric Eye Disease Investigator Group 2004).

The purpose of this study is to investigate how amblyopia treatment is terminated and when patients are being discharged from service; assessing not only the stability of the visual outcome, but also the efficacy and timing of patients’ review.

## Methods

We conducted a retrospective case note review of patients discharged following amblyopia treatment. We included sequential patients who were recently discharged by various orthoptists (five in total) following treatment for strabismic and/or anisometropic amblyopia and had at least two documented follow-up visits. Exclusion criteria were the presence of ocular pathology with visual impact (e.g. cataract or glaucoma surgery), poor attendance (unexplained missing of more than two successive visits or no documented follow up) or poor compliance (failure of part time occlusion or atropine drop instillation as reported by parents and documented in case notes). Patient diagnosis and treatment followed the Guidelines for the Management of Amblyopia set by the Paediatric sub-committee, Royal College of Ophthalmologists (2000), which was observed by all our orthoptists.

Collected data included: Age at the start of treatment, type and duration of treatment, the number of visits and visual acuity (at start and end). Additionally, we analysed the duration and number of visits between cessation of treatment and discharge (end of follow up), along with the final documented visual acuity and duration of treatment with a minimal change in vision. The date of discharge from service was used as an indicator of the full duration of follow up.

Visual acuity was measured and documented using LogMAR and recurrence was identified as visual regression of ≥ 0.2 LogMAR (Nilsson, Baumann and Sjöstrand 2007; Saxena et al. 2013; Walsh et al. 2009; Holmes et al. 2007; Bhola et al. 2006; Pediatric Eye Disease Investigator Group 2004). Descriptive analysis and mean comparison [t-test] was performed on SPSS 17.0 (IBM SPSS Inc., Chicago, IL, USA) using appropriate statistical tests.

This study protocol has gained approval from the local research ethics committee, and data was recorded on the NHS secure folder.

## Results

In the duration between January 2015 to December 2015, 43 sequential patients were discharged from our care; four patients were excluded (two patients had poor compliance to occlusion treatment, one patient had a poor attendance record and one patient had no follow up visits) and 39 patients were included in this study; 19 patients were females and 20 were males. The mean age at start of treatment was 4.2 ± 1.7SD years. The cause of amblyopia was strabismic in 69.2% in cases, anisometropic in 30.8% and none had mixed amblyopia. At the start of treatment eight patients (20.5%) had severe amblyopia (visual acuity > 0.70) and 31 patients (79.5%) had moderate amblyopia (vision 0.20 – 0.60),where the mean difference between the two eyes was 16.4 ± 9.2SD LogMAR (4.1 ± 2.3SD lines).

Treatment was occlusion only in 71.8% and a combination of occlusion and atropine in 28.2%; no patients had atropine alone. With amblyopia treatment 36 patients (92.3%) had improved vision (≥ 0.3 LogMAR); of which 33 patients (84.6%) reached an inter-ocular difference between the treated eye and the sound eye ≤ 0.1 LogMAR, with mean proportion of deficit corrected 0.97 ± 0.3SD. Two patients (5.1%) had no improvement, and one patient (2.6%) had deteriorating vision. The mean letters gained was 13.2 ± 8.7SD and the improvement in vision was statistically significant (t = 9.0, p < 0.005). Mean visual acuities at different stages of care are presented in Table 1.

**Table 1:** Visual acuity at different stages of care.

Visual Acuity at Diagnosis			Visual acuity at the end of treatment			Visual acuity after follow up			Proportion of Deficit Corrected		

*Mean*	*+SD*	*Range*	*Mean*	*+SD*	*Range*	*Mean*	*+SD*	*Range*	*Mean*	*+SD*	*Range*

0.54	0.3	0.15 to 1.20	0.21	0.19	0.05 to 0.90	0.23	0.19	0.00 to 0.90	0.88	0.52	1.2 to –0.05

Most of our patients, 31 (79.5%), had gradual termination of their amblyopia treatment (reducing the duration of patching by half every two weeks) and eight patients (20.5%) were abrupt. Interestingly 11 patients (28.2%), not in the tapering phase, continued to have amblyopia treatment despite having no further improvement in vision. The mean age at termination of amblyopia treatment was 6.9 ± 1.1 years (range 4.2 – 8.7).

Patients were further followed after cessation of treatment for variable durations. Table 2 shows the descriptive statistics of durations (months) and the number of visits while patients were under our care. The mean age after follow up was 7.6 ± 1.4 years (range 4.8 – 11.5).

**Table 2:** Duration and number of visits during different stages of care.

	During active treatment (n = 39)			*With* treatment and *Without* visual improvement (n = 11)			During follow up (n = 39)		

	*Mean*	*SD*	*Range*	*Mean*	*SD*	*Range*	*Mean*	*SD*	*Range*

**Duration (months)**	30.5	21.5	1–89	9.3	11.9	2–64	10	13.5	2–79
**Number of Visits**	16.6	13.9	1–64	5.3	5.6	2–20	5.2	6.6	2–32

Whereas the final visual acuity in the amblyopic eye at discharge remained significantly better than at the start of treatment (t-test; t = 8.9, p < 0.005) there was no significant difference in the vision at the time of termination of treatment and after follow up (t-test; t = 0.42, p = 0.68).

**Figure 1:**
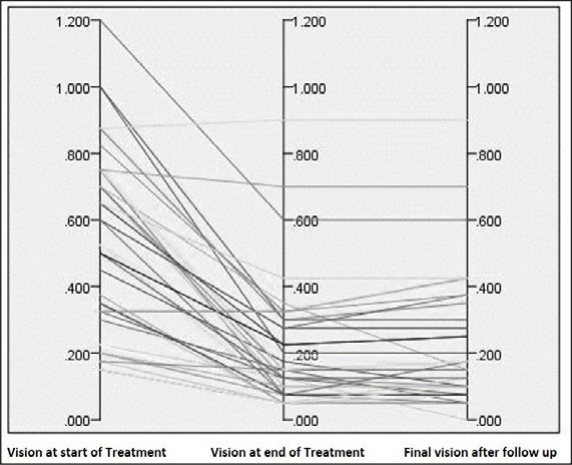
Visual acuity at different stages of care.

No patients had a significant recurrence of their amblyopia (i.e. reduced final visual acuity by ≥ 0.2 LogMAR). However, eight patients (20.5%) showed some reduction in their visual acuity at the time of discharge, which ranged from 0.02 – 0.10 LogMAR (mean 0.059 ± 0.031SD) and no further treatment was deemed necessary by the treating orthoptist. Figure 1 presents the vision of all our patients throughout our care.

Even though none of our patients had significant recurrence of their amblyopia, Table 3 presents previously identified risk factors and how they compared to our cohort along with their incidence in patients with insignificant visual regression (n = 8).

**Table 3:** Published risk factors vs. our data.

Risk Factor		Number of patients (n = 39)	Patients with some visual regression (n = 8)

1.	*Mixed amblyopia*	None (0%)	No patients
2.	*Presence of Microtropia*	6 (15.4%)	1
3.	*Age at start of treatment: > 7 years*	None (0%)	No patients
4.	*Abrupt termination of amblyopia treatment*	8 (20.5%)	1
5.	*Termination of treatment:*		
	*- Before the age of 10 years*	39 (100%)	8
	*- Before the age of 8 years*	37 (94.9%)	5
	*- before the age of 7 years*	20 (51.3%)	3
6.	*Amount of improvement in visual acuity > 0.5 LogMAR*	10 (25.6%)	2
7.	*Final visual acuity: < 0.1 LogMAR*	12 (30.8%)	4

## Discussion

The majority of published studies on the recurrence of amblyopia are prospective with a planned endpoint usually set to one year following termination of amblyopia treatment (Table 4). Whereas most studies have agreed on defining recurrence as visual regression of ≥ 0.2 LogMAR (Nilsson, Baumann and Sjöstrand 2007; Saxena et al. 2013; Walsh et al. 2009; Holmes et al. 2007; Bhola et al. 2006; Pediatric Eye Disease Investigator Group 2004), nevertheless, these studies have not included other meaningful amblyopia outcomes, namely, the percentage of the proportion of the deficit, for which there exists no published pre-set value. Our retrospective study is not in a position to suggest this, therefore we used simple vision regression (≥ 0.2 LogMAR) as our main outcome measure. Current practice lacks evidence-based protocols for termination, follow-up after treatment and robust criteria for patient discharge, rendering it difficult to compare with pre-set research.

**Table 4:** Previous studies on recurrence of amblyopia.

Study	No.	Duration of follow-up	Recurrence rate	Risks

Saxena et al. 2013	102	1 year	12.74%	- Older start (> 7y)
				- Improvement in vision
				- Final vision (< 0.1)
Walsh et al. 2009	52	1 year	24%	- Abrupt termination of treatment
Holmes et al. 2007	69	1 year	25%	- Improvement in vision
				- Final vision
				- Early termination of treatment
Nilsson et al. 2007	35	8 yrs of age	17%	- Microtropia
Tacagni et al. 2007	182	1 year	15%	- Mixed amblyopia
Bhola et al. 2006	653	1 year	27%	- Cessation before ten years
				- Young age at start
PEDIG 2004	145	1 year	24%	- Abrupt termination of treatment

Mean age at diagnosis was 4.2 years and when amblyopia treatment was stopped was 6.9 years, and mean age when patients were discharged was around 7.6 years of age. This is in agreement with published data (Holmes et al. 2007; Pediatric Eye Disease Investigator Group 2004).

Previous studies (Gunton 2013) have documented the effectiveness of amblyopia treatment, and our results support this, with 92.3% improvement in vision (≥ 0.3 LogMAR). However, following visual stability, there was no consistency in termination, follow-up duration or number of visits, and this is evident from the wide spread and range in Table 3.

In the absence of evidence-based guidelines on the final stages of the amblyopia treatment service, the judgment and experience of individual orthoptists decide how long treatment should continue after visual stability and whether termination is gradual or abrupt, in addition to the duration and frequency of follow-up, and timing of discharge from service. In this case series, 28.2% of patients continued treatment not taking place in the tapering phase, for an average of 5.3 visits over 9.3 months with no further improvement of vision; in the absence of termination protocols we cannot justify or disapprove these visits and extra time. Additionally, patients continued under follow-up without treatment with an average of 5.2 visits over 10 months which cannot be compared to prospective studies designed for detecting recurrence of amblyopia; however, there is a general tendency to see patients more frequently over a shorter duration of time.

In comparison to published literature, we had no significant recurrence in amblyopia. A possible explanation could be the inclusion of relatively low risk patients (Table 3) with 0% starting treatment older than 7 years (0.75–6.6 years), 15.4% with microtropia, no patients having mixed amblyopia, 20.5% had abrupt termination of amblyopia treatment, 25.6% had more than 0.5 LogMAR improvements in visual acuity, and 30.8% had a final visual acuity better than 0.1 Log MAR. On the other hand, all of our patients terminated their treatment before the age of 10 years and 51.3% were before the age of 7 years. Furthermore, it seems we did not follow up our patients long enough to accurately confirm our final recurrence rate (average 10 months); this is in contrast to prospective studies which have a pre-set end point at one year (Tacagni et al. 2007; Nilsson, Baumann and Sjöstrand 2007; Saxena et al. 2013; Walsh et al. 2009; Holmes et al. 2007; Bhola et al. 2006; Pediatric Eye Disease Investigator Group 2004).

To summarise: the lack of evidence-based guidelines on the termination of amblyopia treatment has led to the following deficiencies or limitations in this case series:

1. Inconsistent timing of stopping amblyopia treatment; some continued treatment without tapering or improvement of visual acuity
2. Inconsistent method of stopping amblyopia treatment (abrupt vs. tapered)
3. Short and inconsistent duration of follow-up after cessation treatment
4. Inconsistent frequency of follow-up visits after cessation of treatment

Based on these results and evidence from previous publications, there seems to be a need of an agreed termination protocol. The following improvements could be included in future protocols for amblyopia treatment termination:

1. Decision to stop treatment once patients’ have stable vision for three visits 3–4 months apart
2. Amblyopia treatment should be gradually tapered depending on the cause and dose of treatment
3. Patient followed up for two years or up to the age of 9 years old, whichever comes first
4. The frequency of follow-up visits after cessation of treatment could be fixed to four-month intervals

If these suggestions were implemented to our current study, all patients would be followed up without treatment for a maximum of six visits over 24 months. This is three visits less than our current practice average; however, the total follow-up duration will increase from 10 months to 24 months. The average age of discharge will also increase from 7.6 years to 9 years.

## Conclusion

Whereas amblyopia treatment has proven to be effective, it appears there is a large opportunity to improve overall service with regards to the end-point and the number of further visits to ascertain visual stability. An end of treatment protocol is needed to standardise practice and to facilitate future research on visual regression.

This work was accepted for poster presentation at the Royal College of Ophthalmologist Annual Congress, Birmingham, UK, 2016.

## References

[1] Bhola, R, Keech, RV, Kutschke, P, Pfeifer, W and Scott, WE. 2006 Recurrence of amblyopia after occlusion therapy. Ophthalmology, 113: 2097–2100. DOI: 10.1016/j.ophtha.2006.04.034 17074568

[2] Gunton, KB. 2013 Advances in amblyopia: What have we learned from PEDIG trials? Paediatrics, 131: 540–547. DOI: 10.1542/peds.2012-1622 23382445

[3] Holmes, JM, Melia, M, Bradfield, YS, Cruz, OA, Forbes, B, for the Pediatric Eye Disease Investigator Group. 2007 Factors associated with recurrence of amblyopia on cessation of patching. Ophthalmology, 114: 1427–1432. DOI: 10.1016/j.ophtha.2006.11.023 17363058 PMC2384230

[4] Nilsson, J, Baumann, M and Sjöstrand, J. 2007 Strabismus might be a risk factor for amblyopia recurrence. J AAPOS, 11: 240–242. DOI: 10.1016/j.jaapos.2007.01.117 17419081

[5] Pediatric Eye Disease Investigator Group. 2004 Risk of amblyopia recurrence after cessation of treatment. J AAPOS, 8: 420–428. DOI: 10.1016/S1091-8531(04)00161-2 15492733

[6] Pediatric Sub-Committee Group. 2000 Guidelines for the management of strabismus and amblyopia in childhood. Royal College of Ophthalmologists Guidelines.

[7] Saxena, R, Puranik, S, Singh, D, Menon, V, Sharma, P and Phuljhele, S. 2013 Factors predicting recurrence in successfully treated cases of anisometropic amblyopia. Indian J Ophthalmol, 61: 630–633. DOI: 10.4103/0301-4738.123144 24343594 PMC3959076

[8] Tacagni, DJ, Stewart, CE, Moseley, MJ and Fielder, AR. 2007 Factors affecting the stability of visual function following cessation of occlusion therapy for amblyopia. Graefe’s Arch Clin Exp Ophthalmol, 245: 811–816. DOI: 10.1007/s00417-006-0395-2 17047980

[9] Walsh, LA, Erik, K, Hahn, EK and LaRoche, GR. 2009 The method of treatment cessation and recurrence rate of amblyopia. Strabismus, 17(3) 107–116. DOI: 10.1080/09273970903126709 20804358

