# Corrigendum: The Need for a Unified Protocol for Termination of Amblyopia Treatment

**DOI:** 10.22599/bioj.124

**Published:** 2019-01-02

**Authors:** Mahmoud M. Nassar, Fiona Campbell Mitchell

**Affiliations:** 1 Ophthalmology Minia University Hospital, GB; 2 NHS Dumfries and Galloway, GB

**Keywords:** Amblyopia, termination of treatment, recurrent amblyopia

## Abstract

Following the publication of our recent article in British and Irish Orthoptic Journal https://www.bioj-online.com/articles/10.22599/bioj.109/ we wish to bring the following corrigendum to your attention.

## Corrigendum

A typographical error was made in the Results section of the abstract. The abstract should read:

Thirty-nine patients were identified with a mean age 4.2 + 1.7SD years. Patients had an average of 16.6 + 13.9SD visits over 30.5 + 21.5SD months.

The corrected text has been underlined.
